# Nanodiamonds for tracking of leukemic cells

**DOI:** 10.1186/1471-2164-15-S2-P54

**Published:** 2014-04-02

**Authors:** Farid Ahmed, Adnan Memic

**Affiliations:** 1 Center of Excellence in Genomic Medicine, King Abdulaziz University, Jeddah 21589, Saudi Arabia; 2 Center of Nanotechnology, King Abdulaziz University, Jeddah, 21589, Saudi Arabia

## Background

Determination of stem cell behavior *in vivo* is a major challenge in the study of normal and leukemic hematopoiesis. This requires dynamic tracking of cells *in vitro* in cell culture based assays and in vivo in transplantation models. Most of the existing methods for tracking cells are based on gene transfers that might modulate the engraftment and/or leukemogenicity of transplanted cells [1]. In order to evaluate the use of alternative gene-transfer free methods, we tested *in vitro* labeling of leukemic cells using nanodiamonds (ND). NDs have become the most promising candidate in recent years due to their excellent biocompatibility, chemical stability, scalability, fluorescent properties and easy functionalization [2].

## Materials and methods

Human leukemia cell lines HL60 and K562, grown in RPMI1640 medium containing 10% fetal bovine serum, were incubated with different concentrations of 6nm nanodiamond - phosphate buffered saline- suspension. Cell viability was assessed using Trypan blue exclusion method at 24h and 72h of incubation. Flow cytometry was performed after 24h and 72 h of incubation to detect the scatter properties of cells. Confocal fluorescence microscopy was performed to detect nanodiamonds after 24 hr incubation.

## Results

**Figure 1:**
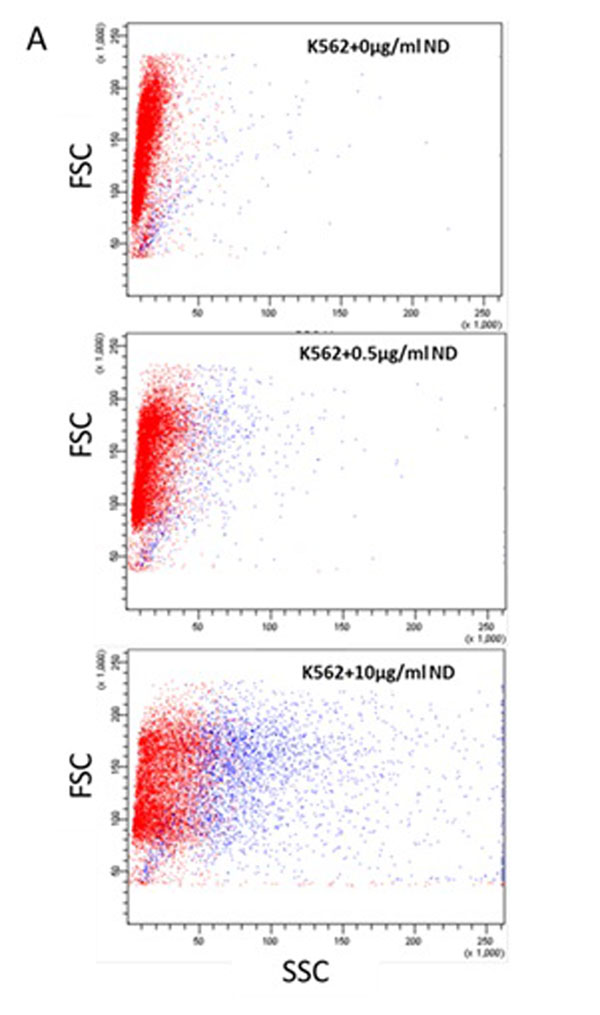
Dose dependent increase in the side scatter of K562 cells incubated with nanodiamonds (ND).

No significant cytotoxicity was observed after incubation of HL60 and K562 cells with up to 10ug/ml ND. Flow cytometry of cells incubated with ND revealed a dose-dependent increase in the side scatter properties of the cells (Figure 1). Confocal microscopy revealed aggregates of fluorescent ND particles in the cytoplasm of both HL60 and K562 cells confirming the uptake of ND. ND+ K562 cells were sorted using the flow cytometer and cultured. ND+ cells were detected up to 5 days post sorting, indicating good retention of these particles

## Conclusions

Our experiments demonstrate for the first time that nanodiamonds can be used successfully in labeling and *in vitro* tracking of leukemic cell lines using flow cytometry and confocal fluorescence imaging, making them a potential candidate for studying *in vivo* tracking in xenograft or syngenic mouse models of leukemia.

*Authors would like to thank KACST for funding the project* (*grant number 09BIO-693-03*)*.*
